# Targeted management of coexistent severe thrombophilias—A case report of a successful pregnancy despite paroxysmal nocturnal hemoglobinuria and hereditary protein C deficiency

**DOI:** 10.1002/jha2.972

**Published:** 2024-07-11

**Authors:** Julien Dereme, Matthew Goodyer, David Baud, Lorenzo Alberio, Francesco Grandoni, Mathilde Gavillet

**Affiliations:** ^1^ Service and Central Laboratory of Haematology Department of Oncology and Department of Laboratories and Pathology Lausanne University Hospital (CHUV) and University of Lausanne (UNIL) Lausanne Switzerland; ^2^ Interregional Blood Transfusion SRC Epalinges Switzerland; ^3^ Service of Haematology Valais Hospital Sion Switzerland; ^4^ Department Woman‐Mother‐Child Lausanne University Hospital University of Lausanne Lausanne Switzerland

**Keywords:** anticoagulation, breakthrough hemolysis, C5‐inhibitor, complement activation, complement inhibitor, eculizumab, paroxysmal nocturnal hemoglobinuria, pregnancy, thrombosis

## Abstract

Paroxysmal nocturnal hemoglobinuria (PNH) is a rare hematological disorder characterized by the absence of complement regulatory proteins on the surface of erythrocytes, leading to intravascular hemolysis and thrombosis. Managing PNH during pregnancy poses significant challenges due to increased risks of morbidity and mortality. This case report describes the detailed obstetric course of a 44‐year‐old woman with PNH and additional hereditary protein C deficiency who had previously experienced multiple thrombotic events and adverse pregnancy outcomes (two early miscarriages and one stillbirth at 25 weeks gestation [WG]), treated with eculizumab (terminal C5 inhibitor) and optimal anticoagulation management. Close monitoring of hemolysis and hemostasis parameters was conducted throughout the gestation period together with increased obstetrical surveillance. The pregnancy progressed without thrombotic complications or breakthrough hemolysis, and the patient delivered a healthy newborn at 36 WG after induction of labor due to restricted fetal growth. To the best of our knowledge, this is the first reported case of a positive pregnancy outcome despite PNH in conjunction with hereditary thrombophilia. This case report highlights the importance of a multidisciplinary approach involving hematologists and obstetricians in the management of pregnant women with PNH. Tailored therapy, close monitoring, and comprehensive care are crucial to minimize risks and optimize outcomes.

## 1 INTRODUCTION

Paroxysmal nocturnal hemoglobinuria (PNH) is characterized by the presence of an acquired somatic mutation of the X‐linked PIGA (phosphatidylinositol glycan‐complementation class A) gene in hematopoietic stem cells, leading to the emergence of a clone lacking the glycosylphosphatidylinositol (GPI) anchor [1]. PNH manifests as a direct antiglobulin test‐negative intravascular hemolysis, resulting in anemia, fatigue, and thrombosis. Thrombophilia is the primary cause of morbidity and mortality in individuals with PNH [2, 3, 4].

Availability of complement inhibitors has revolutionized the management of PNH by controlling intravascular hemolysis, its associated symptoms, and morbidity [5]. Eculizumab, the first developed monoclonal antibody inhibiting C5‐cleavage and preventing the formation of the terminal complement complex C5b‐C9, has demonstrated long‐term safety and efficacy in the treatment of PNH [6, 7, 8].

Despite C5 blockade, managing PNH during pregnancy still poses significant challenges. Pregnant patients with PNH face increased morbidity and a higher risk of foeto‐maternal mortality, primarily due to thromboembolic and infectious complications [9]. Eculizumab has markedly improved the care of pregnant women with PNH, whereas pharmacovigilance analyses have confirmed its safety in this population [8, 10, 11].

Despite these advancements [12, 13], the literature on PNH management during pregnancy remains limited. In this article, we present the case of a patient with PNH and a coexisting hereditary thrombophilia (protein C deficiency) and discuss the management of her pregnancy.

## 2 CASE DESCRIPTION

A 44‐year‐old woman was diagnosed 6 years prior with PNH. She initially presented with dysphagia, abdominal pain, and hemoglobinuria, caused by intravascular hemolysis. Peripheral blood flow cytometry analysis revealed a PNH clone composing 95%, 95%, and 5% of the granulocytes, monocytes, and erythrocytes, respectively. Bone marrow examination ruled out myelodysplastic syndrome or aplasia, and the karyotype was normal (46, female chromosomic caryotype) without any additional mutations detected through oncogenomic analysis. Eculizumab therapy was introduced following meningococcal vaccination, together with folic acid and iron supplement. Since then, the PNH clone remained stable, and hemolysis decreased.

The patient's thromboembolic history includes an ischemic stroke at the age of 21 in 2001, affecting the parietal‐occipital region. An extensive thrombophilia assessment revealed a hereditary deficiency of protein C, with levels at 44% (normal range: 70%–140%) determined by chromogenic assay. Other thrombophilia investigations, including protein S and antithrombin dosages, prothrombin and factor V Leiden mutation gene analysis, and antiphospholipid antibody testing, yielded negative results. PNH was not looked for at the time of the stroke. However, retrospectively, concomitant cytopenia was already consistent with the diagnosis of PNH. Prophylactic direct oral anticoagulant therapy with 10 mg daily rivaroxaban was initiated. At the time of PNH diagnosis, the patient experienced another thrombotic episode, a catheter‐related thrombosis of the cephalic brachial vein. Prophylactic anticoagulation was discontinued 2 weeks after the introduction of eculizumab.

The patient's obstetrical history included two first‐trimester miscarriages and two pregnancy terminations. Moreover, 2 years after PNH diagnosis, she had a new spontaneous pregnancy. Antithrombotic prophylaxis with subcutaneous low molecular weight heparin (LMWH) was started, and eculizumab therapy maintained. The pregnancy was uneventful, until 24.3 weeks gestation (WG) when routine exam showed fetal death. There were no indications of eclampsia or maternal infection. Fetus did not present any dysmorphic features or chromosomal abnormalities. Placenta was underweighted for gestational age and anatomopathological examination revealed numerous infarctions, covering 30%–40% of the surface, along with multiple areas of hypoperfusion.

**FIGURE 1:**
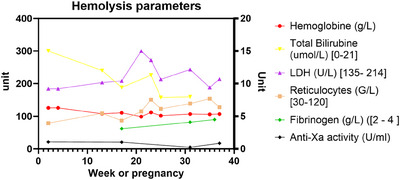
The patient underwent regular clinical follow‐up and hematological monitoring, which did not indicate any episodes of breakthrough hemolysis. Lactate dehydrogenase (LDH) levels remained stable below 1.5 upper limit of the norm, hemoglobin levels ranged from 99 to 112 g/L (normal range: 117–157 g/L), and reticulocyte counts ranged from 79 to 154 g/L (normal range: 20–120 g/L).

After several years of failed conception attempts, the patient had a new pregnancy in 2021 (age 44) following oocyte donation. Therapeutic anticoagulation with LMWH (nadroparin 100 mg q.d. corresponding to 11,400 U.I. anti‐Xa for 60 kg body weight) was started together with antiplatelet therapy with aspirin (100 mg q.d.) according to obstetrical indications (because of previous in utero growth retardation and fetal demise) [14]. Eculizumab therapy was intensified to 1200 mg every 15 days. The patient underwent regular clinical follow‐up and hematological monitoring, which did not indicate any episodes of breakthrough hemolysis. Lactate dehydrogenase (LDH) levels remained stable below 1.5 upper limit of the norm, hemoglobin levels ranged from 99 to 112 g/L (normal range: 117–157 g/L), and reticulocyte counts ranged from 79 to 154 g/L (normal range: 20–120 g/L) (Figure 1). Complement analysis revealed CH‐50 at 0% and sC5b‐9 complex (soluble C5b‐9 bound to S protein/vitronectin [15]) at 207 ng/mL (normal range: 127–303 ng/mL), indicating effective eculizumab therapy. Coagulation parameters showed an increase in d‐dimer levels up to 2337 ng/L, which was outside the expected values for 24 weeks of gestations (291–1231 ng/mL [16, 17]). Residual anti‐fXa activity was always within the therapeutic range (0.2–0.5 U anti‐fXa/mL), and therefore, LMWH dose was not increased. Obstetrical follow‐up, including Doppler indices, demonstrated a healthy pregnancy with normal fetal growth up to 36 WG. Due to sudden absence of growth over the last 2 weeks, maternal age, previous in utero death and to optimize peripartum anticoagulation management, labor was induced at 36 WG. The baby weighted 2660 g and had an uneventful neonatal follow‐up. Aspirin had been stopped 2 weeks prior, and LMWH was switched 24 h before induction to therapeutic intravenous unfractionated heparin to minimize the therapeutic window and reduce the thromboembolic risk during the peripartum period. The patient had an uncomplicated vaginal delivery (blood losses <500 mL), and the newborn was healthy. Therapeutic LMWH was immediately reintroduced for 6 more weeks postpartum. Eculizumab was decreased to the usual dose of 900 mg and maintained during breastfeeding prior to switching to ravulizumab. No complications were reported during postpartum and breastfeeding. The histological study of the placenta at birth revealed a villitis of undetermined etiology; however, the vascular inspection showed vessels of regular caliber and distribution without signs of ischemia, thrombosis, or infarction.

## 3 DISCUSSION

To the best of our knowledge, this is the first case report of a positive pregnancy outcome despite PNH in conjunction with hereditary thrombophilia. Assessing the true thrombotic risk in such an intricate situation is difficult due to the interplay of various factors acting on primary, secondary as well as tertiary hemostasis. With appropriate management of each component, the pregnancy proceeded without any thrombotic complications nor breakthrough hemolysis, and outcome was favorable for both mother and child.

Prior to the introduction of C5‐inhibitors, transfusions were the only treatment for the anemia of PNH. Unaddressed, the prothrombotic effect of hemolysis was associated with a high incidence of venous thromboembolism during pregnancy (10%) and decreased life‐expectancy [9], leading to pregnancy being discouraged in these patients. Eculizumab has demonstrated its efficacy in preventing complications of PNH in general as well as during pregnancy. Kelly et al. reported favorable outcomes during pregnancy in 61 women with PNH receiving eculizumab, with a high rate of live births and a low rate of maternal complications [13]. Complement activation increases during pregnancy, particularly after 20 weeks [18], and breakthrough hemolysis is common. Due to the physiological changes that occur during pregnancy (increased distribution volume, enhanced lysosomal activity), all patients require terminal complement inhibition and if previously under such therapy, most require an increased dose and/or frequency of eculizumab. However, there is no international consensus yet; some authorities argue for preventive dose escalation at the end of the first trimester, whereas others advise close clinical and biological monitoring with a low‐threshold for dose escalation. Considering the dramatic course of the previous pregnancy, our patient received an increased dose of 1200 mg intravenously every 15 days as soon as possible. This management strategy successfully prevented breakthrough hemolysis, as evidenced by the stable levels of LDH, hemoglobin, and reticulocyte count throughout gestation. Monitoring complement activation by following classical pathway activation CH50 and activation products sC5b‐9 serves as a useful marker to ensure efficient complement inhibition. The safety of eculizumab is well‐established, as it does not pass into fetal blood or breast milk at concentrations concerning for fetal complement inhibition [13]. Presently, these data are lacking regarding other complement inhibitors, and C5‐blockade remains the mainstay of PNH therapy during pregnancy and lactation.

Pregnancy constitutes a prothrombotic state that culminates in the first weeks postdelivery, due to increased coagulations factor such as von Willebrand and factor VIII, decreased natural anticoagulants, and decreased fibrinolysis. The decrease in peripartum blood loss comes at the expense of increased thrombo‐embolic events about 2–5 times higher than age‐ and gender‐matched counterparts [19]. PNH increases thrombotic risk due to platelet activation, complement‐mediated hemolysis, impaired nitric oxide bioavailability, impairment of the fibrinolytic system by lack of GPI anchored protein, endothelial dysfunction, and inflammatory mediators [20]. After activation by thrombomodulin‐bound thrombin, protein C downregulates coagulation by cleaving and inactivating clotting factors Va and VIIIa. Individually, PNH and hereditary thrombophilia constitute an indication for thromboprophylaxis during pregnancy and the 6 weeks postpartum [21]. Most authors agree that all pregnant patients with PNH and a history of thrombosis should receive therapeutic anticoagulation. For our patient, given the presence of a previous arterial stroke, along with hereditary protein C deficiency and the hypercoagulable state of PNH, there was a clear indication for full‐dose anticoagulation during pregnancy. Monitoring d‐dimer and fibrinogen levels may help identify patients at increased thrombotic risk who could benefit from increased‐intensity anticoagulation [16, 17]. In this case, the patient received full‐dose anticoagulation, and the effect on hemostatic parameters remained stable without any signs of hemorrhagic diathesis including at delivery.

## 4 CONCLUSION

PNH poses significant risks for both the fetus and the mother. Preconception planning, a multidisciplinary approach, close clinical, biological and ultrasonographic monitoring are all key components to minimizing the risks and optimizing the management of this complex condition throughout pregnancy. Several factors need to be closely monitored and managed in PNH patients during pregnancy, including hemolysis and hemostasis parameters, the need for increased doses of eculizumab, initiation or adjustment of anticoagulation therapy, the degree of bone marrow failure, and the immunosuppressed status of the patient. Additionally, special attention should be given to the presence of other associated thrombotic conditions or thrombophilia. With the combined expertise of hematologists and obstetricians, comprehensive care can be provided to pregnant women with PNH, minimizing the risks and optimizing the management of this complex condition throughout the pregnancy.

## AUTHOR CONTRIBUTIONS

*Writing—original draft preparation*: Julien Dereme and Mathilde Gavillet. *Writing—review and editing*: Julien Dereme, Matthew Goodyer, Lorenzo Alberio, Francesco Grandoni and Mathilde Gavillet.

## CONFLICT OF INTEREST STATEMENT

All the authors (J.D., M.G., L.A., F.G., M.G.) in this case report declare no conflicts of interest.

## FUNDING INFORMATION

This research did not receive any specific grant from funding agencies in the public, commercial, or not‐for‐profit sectors.

## ETHICS STATEMENT

The authors have confirmed that ethical approval statement is not needed for this submission.

## PATIENT CONSENT STATEMENT

The authors have confirmed patient consent statement is not needed for this submission.

## PERMISSION TO REPRODUCE MATERIAL FROM OTHER SOURCES

NA.

## CLINICAL TRIAL REGISTRATION (INCLUDING TRIAL NUMBER)

The authors have confirmed that clinical trial registration is not needed for this submission.

## Data Availability

The data that support the findings of this study are available from the corresponding author upon reasonable request.

## References

[1] Hill A , De Zern AE , Kinoshita T , Brodsky RA . Paroxysmal nocturnal haemoglobinuria. Nat Rev Dis Primers. 2017;3:17028. 10.1038/nrdp.2017.2810.1038/nrdp.2017.28 28516949 PMC7879566

[2] Parker C , Omine M , Richards S , Nishimura J , Bessler M , Ware R , et al. Diagnosis and management of paroxysmal nocturnal hemoglobinuria. Blood. 2005;106(12):3699–3709. 10.1182/blood-2005-04-1717 16051736 PMC1895106

[3] Hillmen P , Lewis SM , Bessler M , Luzzatto L , Dacie JV . Natural history of paroxysmal nocturnal hemoglobinuria. N Engl J Med. 1995;333(19):1253–1258. 10.1056/NEJM199511093331904 7566002

[4] Socié G , Mary JY , de Gramont A , Rio B , Leporrier M , Rose C , et al. Paroxysmal nocturnal haemoglobinuria: long‐term follow‐up and prognostic factors. French Society of Haematology. Lancet. 1996;348(9027):573–577. 10.1016/s0140-6736(95)12360-1 8774569

[5] Rother RP , Rollins SA , Mojcik CF , Brodsky RA , Bell L . Discovery and development of the complement inhibitor eculizumab for the treatment of paroxysmal nocturnal hemoglobinuria. Nat Biotechnol. 2007;25(11):1256–1264. 10.1038/nbt1344 17989688

[6] Brodsky RA , Young NS , Antonioli E , Risitano AM , Schrezenmeier H , Schubert J , et al. Multicenter phase 3 study of the complement inhibitor eculizumab for the treatment of patients with paroxysmal nocturnal hemoglobinuria. Blood. 2008;111(4):1840–1847. 10.1182/blood-2007-06-094136 18055865

[7] Hillmen P , Muus P , Röth A , Elebute MO , Risitano AM , Schrezenmeier H , et al. Long‐term safety and efficacy of sustained eculizumab treatment in patients with paroxysmal nocturnal haemoglobinuria. Br J Haematol. 2013;162(1):62–73. 10.1111/bjh.12347 23617322 PMC3744747

[8] Socié G , Caby‐Tosi MP , Marantz JL , Cole A , Bedrosian CL , Gasteyger C , et al. Eculizumab in paroxysmal nocturnal haemoglobinuria and atypical haemolytic uraemic syndrome: 10‐year pharmacovigilance analysis. Br J Haematol. 2019;185(2):297–310. 10.1111/bjh.15790 30768680 PMC6594003

[9] Ray JG , Burows RF , Ginsberg JS , Burrows EA . Paroxysmal nocturnal hemoglobinuria and the risk of venous thrombosis: review and recommendations for management of the pregnant and nonpregnant patient. Haemostasis. 2000;30(3):103–117. 10.1159/000022532 11014960

[10] Sarno L , Tufano A , Maruotti GM , Martinelli P , Balletta MM , Russo D . Eculizumab in pregnancy: a narrative overview. J Nephrol. 2019;32(1):17–25. 10.1007/s40620-018-0517-z 30159857

[11] Miyasaka N , Miura O , Kawaguchi T , Arima N , Morishita E , Usuki K , et al. Pregnancy outcomes of patients with paroxysmal nocturnal hemoglobinuria treated with eculizumab: a Japanese experience and updated review. Int J Hematol. 2016;103(6):703–712. 10.1007/s12185-016-1946-x 26857155

[12] de Guibert S , Peffault de Latour R , Varoqueaux N , Labussière H , Rio B , Jaulmes D , et al. Paroxysmal nocturnal hemoglobinuria and pregnancy before the eculizumab era: the French experience. Haematologica. 2011;96(9):1276–1283. 10.3324/haematol.2010.037531 21606169 PMC3166097

[13] Kelly RJ , Höchsmann B , Szer J , Kulasekararaj A , de Guibert S , Röth A , et al. Eculizumab in pregnant patients with paroxysmal nocturnal hemoglobinuria. N Engl J Med. 2015;373(11):1032–1039. 10.1056/NEJMoa1502950 26352814

[14] Loussert L , Vidal F , Parant O , Hamdi SM , Vayssiere C , Guerby P . Aspirin for prevention of preeclampsia and fetal growth restriction. Prenat Diagn. 2020;40(5):519–527. 10.1002/pd.5645 31955436

[15] Chiu YY , Nisihara RM , Würzner R , Kirschfink M , de Messias‐Reason IJ . SC5b‐9 is the most sensitive marker in assessing disease activity in Brazilian SLE patients. J Invest Allergol Clin Immunol. 1998;8(4):239–244. 9777539

[16] Siennicka A , Kłysz M , Chełstowski K , Tabaczniuk A , Marcinowska Z , Tarnowska P , et al. Reference values of D‐dimers and fibrinogen in the course of physiological pregnancy: the potential impact of selected risk factors‐A pilot study. Biomed Res Int. 2020;2020:3192350. 10.1155/2020/3192350 32596295 PMC7273490

[17] Chabloz P , Reber G , Boehlen F , Hohlfeld P , de Moerloose P . TAFI antigen and d‐dimer levels during normal pregnancy and at delivery. Br J Haematol. 2001;115(1):150–152. 10.1046/j.1365-2141.2001.03082.x 11722426

[18] Derzsy Z , Prohászka Z , Rigó J Jr , Füst G , Molvarec A . Activation of the complement system in normal pregnancy and preeclampsia. Mol Immunol. 2010;47(7–8):1500–1506. 10.1016/j.molimm.2010.01.021 20181396

[19] DeLoughery E , Bannow BS . Anticoagulant therapy for women: implications for menstruation, pregnancy, and lactation. Hematol Am Soc Hematol Educ Program. 2022;2022(1):467–473. 10.1182/hematology.2022000401 PMC9820577 36485151

[20] Hill A , Kelly RJ , Hillmen P . Thrombosis in paroxysmal nocturnal hemoglobinuria. Blood. 2013;121(25):4985–4996. 10.1182/blood-2012-09-311381 23610373

[21] Bates SM , Rajasekhar A , Middeldorp S , McLintock C , Rodger MA , James AH , et al. American Society of Hematology 2018 guidelines for management of venous thromboembolism: venous thromboembolism in the context of pregnancy. Blood Adv. 2018;2(22):3317–3359. 10.1182/bloodadvances.2018024802 30482767 PMC6258928

